# Family Cohesion and Sleep Disturbances During COVID-19: the Mediating Roles of Security and Stress

**DOI:** 10.1007/s11469-022-00753-w

**Published:** 2022-02-07

**Authors:** Baojuan Ye, Jing Hu, Hohjin Im, Mingfan Liu, Xinqiang Wang, Qiang Yang

**Affiliations:** 1 Center of Mental Health Education and Research, School of Psychology, Jiangxi Normal University, 99 Ziyang Avenue, Nanchang, 330022 China; 2 Department of Psychological Science, University of California Irvine, 4201 Social & Behavioral Sciences Gateway, Irvine, CA 92697 USA; 3 School of Education, Jiangxi Normal University, 99 Ziyang Avenue, Nanchang, 330022 China

**Keywords:** COVID-19, Perceived stress, Sleep disturbances, Family cohesion, Sense of security

## Abstract

Despite the increase in proximity to one's family amid university closures during the COVID-19 pandemic, the mechanisms underlying how family cohesion influenced students’ sleep remain understudied. Using a large sample of college students in China (*N* = 1,178) during the COVID-19 pandemic, the current study examined the serial mediating roles of sense of security and perceived stress on the effect of family cohesion on sleep disturbance. Generalized linear modeling serial mediation analysis with 1,000 resampled bootstrapping methods showed that sense of security and perceived stress were negatively and positively associated with sleep disturbance, respectively. Furthermore, sense of security and perceived stress fully mediated the indirect effect of family cohesion on sleep disturbances. Implications and conclusions are discussed.

## Introduction

For many college students, the COVID-19 pandemic presented a novel threat to their psychological well-being. This particularly rang true for college students in China during the early months of 2020 as the novel coronavirus ravaged neighboring provinces and the trajectory of the pandemic was unclear. Expectedly, many scholars advocated for greater understanding of how sleep was impacted early in the pandemic (Gupta et al., 2020; Huang & Zhao, 2020; Pinto et al., 2020; Sher, 2020). Despite the abundance of studies on COVID-19 and sleep, however, literature on the sleep disturbances of college students in China has not been commensurate with the heightened risk to their mental health amid their changing social environment.

In early 2020, China was the first country to implement a government-mandated lockdown and extend winter recess for many universities in an effort to curb the viral spread of COVID-19. Although it is common for Chinese universities to observe reduced foot traffic during holidays as students return to their families (McCarthy, 2020), this was particularly pronounced during the COVID-19 pandemic. The consequences of prolonged proximity to one’s family has since become subject to scholarly scrutiny (Prime et al., 2020). Recent studies have shown both troubling revelations of increased family violence (Zhang, 2020) and positive signs of reduced depressive symptomology from familial support (Mariani et al., 2020). However, the impact of familial environment on sleep disturbances has received relatively little attention from researchers both during this pandemic and also historically (Billows et al., 2009) in spite of epidemiological evidence of its key role in shaping the sleeping patterns of youth (e.g., Johnson et al., 2018).

Sleep disturbance refers to the subjective dissatisfaction with one’s sleep duration or quality, which subsequently negatively affects daily social functioning (Li et al., 2006). As a key antecedent of physical and mental health (Vazsonyi et al., 2018), understanding the correlates of sleep disturbance among students in China may elucidate important mechanisms for designing needed interventions for vulnerable youth who may otherwise not be well equipped to handle the psychological burdens in facing a global pandemic. Thus, the current study examines how family cohesion may be associated with outcomes of security and stress that, in turn, may explain individual variability in experiencing sleep disturbances.

### Family Cohesion and Sleep Disturbances

Family cohesion refers to the emotional bonding and interdependence of family members—the degree to which individuals are accepted in the family system and can rely on their family members for support (Olson, 1986). A primary function of families is to promote the positive growth of youth (Aydin & Oztutuncu, 2001; Gardner & Cutrona, 2004). Positive familial environments protect at-risk youths from negative environmental influences (Kliewer et al., 2006; Qin et al., 2015) and subsequent development of maladaptive psychological and behavioral outcomes by providing a foundation for fostering interpersonal security (Cummings et al., 2015; Ye, Lei et al., 2021; Ye, Fan, et al., Working Paper). *Maslow’s hierarchy of needs* holds that a sense of security is the subjective cognition and reaction to one’s own security state separated from anxiety and fear (Cong & An, 2004; Maslow et al., 1945). An individual who loses their basic experience of security may also suffer a subsequent reduction in their life satisfaction, likely exacerbating additional negative emotional problems (Tang et al., 2018). Thus, we posit the following:

- Hypothesis 1a: Family cohesion is positively related to sense of security.

In a similar vein, another primary function of families is to be a source of social support, aiding in the development of youths’ individual emotional adaptation and stress coping (Bannink et al., 2013; de Haymes et al., 2011). The Family Stress model posits that disruptions to basic family functioning, such as distressed parenting precedes several maladaptive childhood and adolescent outcomes (Masarik & Conger, 2017). On the other hand, families capable of adequately providing social support are able to mitigate stress contagion (Tsai et al., 2018) and promote adaptive coping (Rojo-Wissar et al., 2020; Sadeh et al., 2000). In other words, family cohesion may serve as a protective factor against the experience of subjective stress stemming from interpersonal conflicts and external issues (Coe et al., 2017; Masarik & Conger, 2017; Ye, Fan, et al., Working Paper). Thus, we posit the following hypothesis:

- Hypothesis 1b: Family cohesion is negatively related to perceived stress.

Despite the benefits of positive familial environments, however, family cohesiveness varies greatly. In direct contrast to cohesive families, dysfunctional family units are ill-equipped to adequately promote children's proper development of sleeping habits owing to its inherent disorderly state (Billows et al., 2009; Peltz et al., 2019; Spilsbury et al., 2017). Parents in incohesive units frequently fail to function as role models that exemplify and enforce proper sleep hygiene among children and adolescents who are more dependent on their parents to learn and mirror social development (Cousins et al., 2007; Gregory et al., 2005). In the absence of familial order, youth are at greater risk of experiencing sleep disturbances and exhibiting insomnia symptomology (Billows et al., 2009; Gregory et al., 2005, 2006). On the other hand, positive family factors are associated with better sleep quality among adolescents and youth (Rojo-Wissar et al., 2020; Sadeh et al., 2000; Tsai et al., 2018). Particularly during times of a global pandemic, where a myriad of stressors may be common, family cohesion may therefore be a key antecedent in mitigating sleep disturbances:

- Hypothesis 1c: Family cohesion is negatively related to sleep disturbances.

### Sense of Security as a Mediating Mechanism

Family cohesion’s link to security is likely to have notable downstream implications. The COVID-19 pandemic may be construed as an optimal breeding ground for promoting psychological insecurity and stress; prior evidence of crisis-related negative life events (e.g., emergencies, earthquakes, infectious diseases) have foreshadowed an increase in negative emotions (e.g., anxiety, panic) associated with the deterioration of one’s sense of security (e.g., Buzohre et al., 2018). Although the various preventive measures (e.g., forced closures of college campuses) may be epidemiologically warranted, these actions may also serve as cues to legitimize the impending threat of the virus and the loss of one’s agentic control and security over their environment. Accordingly, the loss of psychological security may increase stress levels and deteriorate sleep quality among those affected.

The theory of sleep disturbance process posits that negative psychological states will interfere with normal sleep processes and negatively influence sleep quality (Jin et al., 2011). Indeed, prior studies have routinely linked sleep quality with attachment style (Adams et al., 2014), interpersonal distress (Gunn et al., 2014), and exposure to interpersonal violence (Berg et al., 2021) stemming from exacerbated levels of negative emotional and psychological arousal. In other words, the heightened sense of insecurity may leave one susceptible to the detriments of negative thoughts and emotions, producing a vicious cycle of despondency that increases disturbances to sleep (Roth et al., 2019). Thus, the following hypotheses are given:

- Hypothesis 2a: Sense of security is negatively related to stress.
- Hypothesis 2b: Sense of security is negatively related to sleep disturbance.

### Perceived Stress and Sleep Disturbances

Stress has long been evidenced to be one of the fundamental drivers in the onset and development of sleep issues (e.g., Amaral et al., 2018; Benham & Charak, 2019; Brand et al., 2011; Gerber et al., 2010; Lee et al., 2013; Yan et al., 2010). However, the extent to which stress influences an individual is subject to its perceived magnitude (e.g., Lazarus & Folkman, 1984). Perceived stress not only impacts one’s cognitive functioning but also their emotional and physiological state (Lazarus & Folkman, 1984), including disruptions to sleep (Yan et al., 2010) stemming from heightened emotional arousal states (Sterling et al., 1988). Consequently, negative effect of perceived stress on sleep quality has been pervasive (Amaral et al., 2018; Benham & Charak, 2019) and robust during the COVID-19 pandemic (Gupta et al., 2020; Huang & Zhao, 2020; Pinto et al., 2020).

As China was the site of the first outbreak of COVID-19 cases, college students in China were among the first set of youth to have been affected by the pandemic. During China’s first wave of COVID-19, college students reported a diverse range of stress sources, such as social groups, media, and school closures (Wang & Zhao, 2020; Ye, Wu, et al., 2020; Ye, Zhou, et al., 2020; Ye, Yang, et al., 2020; Z. Ye et al., 2020; Ye, Zhou, et al., 2020; Ye, Yang, et al., 2020). Correspondingly, students reported increased tendencies in maladaptive behaviors, such as rumination and, in addition to experiencing negative psychological outcomes (Ye, Wu et al., 2020; Ye, Zhou, et al., 2020; Ye, Lei, et al., 2021; Ye, Zeng, et al., 2021; Ye, Yang, et al., 2020). The backlog of these series of psychological burdens may explain individual variability in sleep disturbances (Li et al., 2019; Ye, Wu, et al., 2021). Thus, we posit the following hypothesis:

- Hypothesis 3: Perceived stress is positively related to sleep disturbances.

**Fig. 1:**
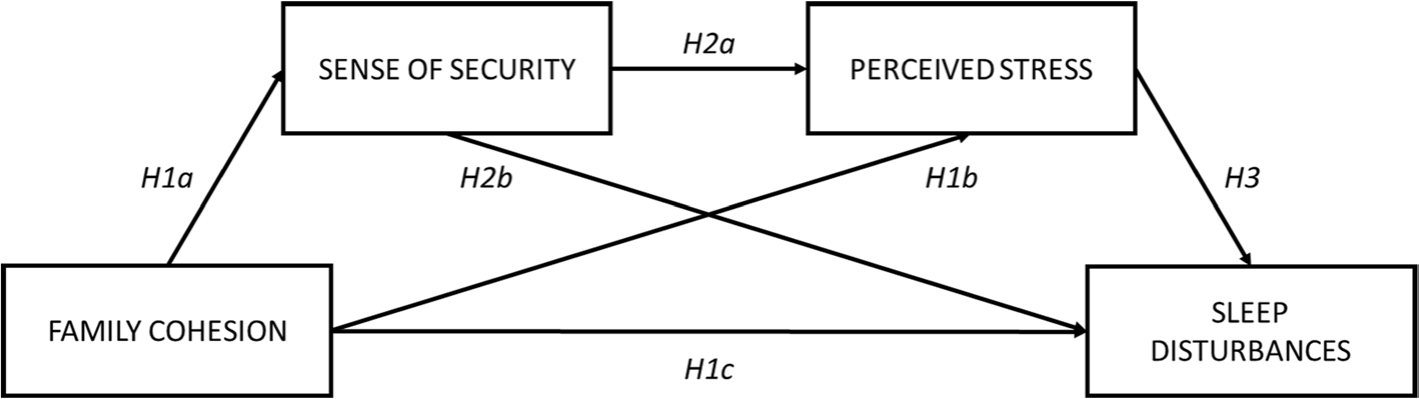
Conceptual mediation model

Lastly, based on the given literature, each discussed construct of family cohesion, security, and stress conceptually precede the following in their shared link to sleep disturbances. In other words, we posit that the association between these variables in their explanation of individual variability of sleep disturbances may be conceptually modeled in as a serial mediation (Fig. 1) with the following hypothesis:

- Hypothesis 4: Sense of security and perceived stress mediate the effect of family cohesion on sleep disturbances.

## Method

### Participants

A total of 1,178 college students (age *M* = 20.115, *SD* = 1.403, 68.3% female) in China participated in this study. The sample consisted of 43.5% first years, 21.3% second years, 26.7% third years, and 8.5% fourth years or higher.

### Procedures

The current study was approved by the Ethics Committee of the first author’s university. All study participants provided informed consent before being directed to an anonymous online survey in which they completed the measures listed in the following section.

### Measures

#### Perceived Stress

Perceived stress was measured via the Perceived Stress Scale (Cohen, Kamarch, & Mermelstein, 1983), Chinese version revised by Yang and Huang (2003). This scale consisted of 14 items (e.g., “Getting upset when something unexpected happens”) and included two dimensions: (1) nervousness (7 items) and (2) perceived control (7 items), *ω* = 0.789. Each item was rated on a 5-point scale (1 = *never* to 5 = *always*), with higher total scores indicating higher levels of perceived stress.

#### Sense of Security

Sense of security was measured via the Sense of Security Scale (Maslow et al., 1945), Chinese version revised by Cong and An (2004). This scale consisted of 16 items (e.g., “I’m afraid to establish and maintain an intimate relationship with others”) and included two dimensions: (1) interpersonal security (8 items) and (2) sense of certainty and control over future life (8 items), *ω* = 0.943. Each item was rated on a 5-point scale (1 = *absolutely the same* to 5 = *very different*), with higher total scores indicating higher levels of sense of security.

#### Family Cohesion

Family cohesion was measured via a shortened version of the Family Cohesion Scale (Olson et al., 1982), Chinese version revised by Fei et al. (1991), which consisted of 12 items (e.g., “Family members are familiar with each other’s close friends”), *ω* = 0.938. Each item was rated on a 5-point scale (1 = *never* to 5 = *always*), with higher total scores indicating higher levels of family cohesion.

#### Sleep Disturbances Scale

Sleep disturbances were measured via the shortened Chinese adaptation version (Liu et al., 1996of the Pittsburgh Sleep Quality Scale (Buysse et al., 1989). The scale included 13 items (e.g., “Over the past half month, how is your overall sleep quality”). This scale consists of three dimensions: (1) subjective sleep quality (three items), (2) sleep disorder (six items), and (3) daytime dysfunction (four items), *ω* = 0.917. Each item was rated on a 4-point scale assessing one’s dissatisfaction (1 = *Very Satisfied* to 4 = *Very Dissatisfied*), frequency (1 = *Never* to 4 = 3–5 *times a week*), duration (1 = *More than 8 h* to 4 = *Less than 5 h*), and reoccurrence (1 = *Not at all* to 4 = *Almost every day*), with higher total scores indicating higher levels of sleep disturbances.

### Data Analysis

All variables were standardized and showed no significant deviation from normal distribution (skew from − 0.316 to 0.879, kurtosis from 0.003 to 0.480) (Hancock & Mueller, 2010). The serial mediation model was analyzed using generalized linear modeling with 1,000 resamples of parametric bootstrap confidence intervals (CIs).

## Results

### Descriptive Analyses

Descriptive and bivariate correlation statistics are reported in Table 1. Sleep disturbance was positively correlated with perceived stress while negatively correlated with both sense of security and family cohesion. Perceived stress was likewise negatively correlated with both sense of security and family cohesion. Sense of security was positively related to family cohesion.

**Table 1:** Descriptive statistics and correlations

Predictors	M	SD	1	2	3	4
Sleep disturbances	1.852	0.664	—			
Perceived stress	2.657	0.480	0.451	—		
Sense of security	3.505	0.710	− 0.449	− 0.528	—	
Family cohesion	3.286	0.748	− 0.154	− 0.416	0.195	—

Note: all correlations significant at *p* < 0.001

### Mediation Effect of Sense of Security

Regression analyses (Table 2) showed that family cohesion was positively related to sense of security (model 2) and negatively related to perceived stress (model 3). Family cohesion was only directly associated with sleep disturbance when examined in isolation (model 1) but not when controlling for security or stress (model 4, *p* = 0.226). Sense of security was negatively associated with both perceived stress (model 3) and sleep disturbance (model 4). Perceived stress remained a strong positive correlate of sleep disturbance even after controlling for all other variables (model 4). All models showed no sign of multicollinearity (VIF from 1.00 to 1.62). Post-hoc power analysis also indicated all models were sufficiently powered (0.999 to 1.000).

**Table 2:** Regression paths of the conceptual model

Predictors	Model 1	Model 2	Model 3	Model 4
	Sleep Dist	Security	Stress	Sleep Dist
	β (95% CI)	β (95% CI)	β (95% CI)	β (95% CI)
Intercept	− 0.086	0.116 *	− 0.066	− 0.015
	(− 0.187, 0.015)	(0.016, 0.216)	(− 0.147, 0.014)	(− 0.103, 0.073)
Female	0.126 *	− 0.170 **	0.097	0.022
	(0.003, 0.248)	(− 0.291, − 0.049)	(− 0.001, 0.195)	(− 0.085, 0.128)
Age	0.024	0.014	− 0.022	0.038
	(− 0.033, 0.081)	(− 0.042, 0.071)	(− 0.068, 0.023)	(− 0.012, 0.087)
Family cohesion	− 0.153 ***	0.195 ***	− 0.327 ***	0.033
	(− 0.210, − 0.097)	(0.139, 0.251)	(− 0.373, − 0.281)	(− 0.021, 0.087)
Sense of security			− 0.460 ***	− 0.291 ***
			(− 0.506, − 0.414)	(− 0.349, − 0.233)
Perceived stress				0.312 ***
				(0.249, 0.374)
Observations	1178	1178	1178	1178
*R*^2^/*R*^2^ adjusted	0.027/0.025	0.045/0.042	0.384/0.382	0.268/0.264

Note: ^*^p < 0.05, ^**^p < 0.01, ^***^p < 0.001; *Sleep Dist*., sleep disturbances

### Serial Mediation Analysis

**Fig. 2:**
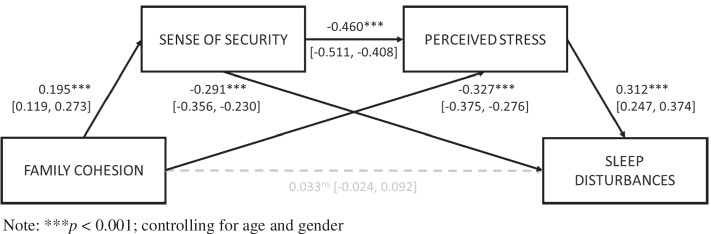
Generalized linear model serial mediation

Mediation analyses with 1,000 bootstrapped resampling (Fig. 2) showed that consistent with regression analyses of each individual path, sense of security and perceived stress serially mediated the association between family cohesion and sleep disturbance (*β* =  − 0.028, *z* =  − 4.23, 95% CI [− 0.041, − 0.015], *p* < 0.001). No direct effect of family cohesion on sleep disturbance was found (*β* = 0.033, *z* = 1.13, 95% CI [− 0.024, 0.092], *p* = 0.259) but was individually mediated by both sense of security (*β* =  − 0.057, *z* =  − 4.509, 95% CI [− 0.082, − 0.033], *p* < 0.001) and perceived stress (*β* =  − 0.102, *z* =  − 7.6, 95% CI [− 0.127, − 0.075], *p* < 0.001).

## Discussion

Although the link between perceived stress and sleep disturbances has been extensively studied over the years, there has been little inquiry into how this effect may be preceded by variables relevant to the social ecological developments during the early stages of the pandemic. In this study, a serial mediation model showed how the relation between family cohesion and sleep disturbances was funneled through sense of security and perceived stress.

### Family Cohesion’s Role in Sleep Disturbances

General linear modeling showed that family cohesion was positively associated with sense of security and negatively associated with perceived stress, consistent with recent research examining the outcomes of family cohesiveness during the pandemic (Ye, Lei, et al., 2021; Ye, Zeng, et al., 2021; Ye, Fan, et al., Working Paper). Although family cohesion was initially directly correlated with sleep disturbances, consistent with prior studies (Rojo-Wissar et al., 2020; Sadeh et al., 2000; Tsai et al., 2018), its association became nonsignificant upon the inclusion of security and stress. In other words, it is likely that any interventions targeting family cohesion (e.g., family therapy) would see downstream effects—rather than direct ones—on improving sleep quality.

Family cohesion has been posited to be associated with a multitude of different well-being outcomes across development (Crespo et al., 2011; Mitchell et al., 2016). Although the current investigation’s use of cross-sectional data limits the ability to make causal inferences, should the proposed conceptual model accurately reflect latent causal paths, family cohesion may be a vital antecedent to intervene on. Although understudied, several studies have found positive implications of family therapy on improving family cohesiveness and adolescent mental health (e.g., Bozorgmanesh et al., 2016; Cumsille & Epstein, 1994). Such endeavors may be particularly important in curbing long-term negative mental health outcomes and the development of sleep disorders among youth who were negatively affected during the COVID-19 pandemic.

### Sense of Security’s Role in Sleep Disturbances

Sense of security was strongly and negatively associated with both stress and sleep disturbances. The negative path from sense of security to stress may partly be a reflection of college students’ variability in becoming more acutely aware of their own health conditions in the presence of a largely uncontrollable global pandemic. That is, with heightened awareness and attention to one’s vulnerability, students with a lower sense of psychological security may experience a greater panic or wariness (Ayittey et al., 2020). Furthermore, an insecure psychological state may have made students vulnerable to a myriad of negative cognitive thoughts, such as rumination (Ye, Wu, et al., 2020; Ye, Zhou, et al., 2020; Ye, Yang, et al., 2020). From the perspective of the cognitive model of sleep disturbances (Harvey, 2002), the outbreak of the COVID-19 may dispose college students lacking security to experience pervasive negative states that inhibits quality sleep (Li et al., 2019).

It is worth noting, however, that perceived stress only partially mediated the relation between sense of security and sleep disturbances, suggesting that sense of security remains a prominent, direct protective factor against sleep disturbances among college students. For students with a lower sense of security, the accompanying negative emotions and thoughts may be pervasive obstacles in maintaining normative daily functioning. This maladaptive cognitive tendency can aggravate additional negative assessments and behavioral tendencies (e.g., emotional suppression, low self-esteem) that may lead to a decline in the sleep quality (Donarelli et al., 2016; Johnson et al., 2006). Nonetheless, those able to cognitively reappraise their situations fare better in improving their sleep quality (Ye, Wu, et al., 2021) and mindset interventions may aid in improving psychological security and coping.

### Stress’s Role in Sleep Disturbances

The positive relation between stress and sleep disturbances was an expected finding in the grim story continuously being expanded in the health and clinical scholarships. The finding is consistent with research prior to the pandemic (Amaral et al., 2018; Benham & Charak, 2019; Brand et al., 2011; Gerber et al., 2010; Lee et al., 2013; Yan et al., 2010) as well as those during the pandemic (Huang & Zhao, 2020; Ye, Wu, et al., 2020; Ye, Lei, et al., 2021; Ye, Wu, et al., 2021; Ye, Yang, et al., 2020). In other words, although the current model’s measure of stress was a generic one—and not specific to COVID-19—it is likely that one’s perceived general stress also overlaps with COVID-19 stressors. In the current study, family cohesion and sense of security were also both mediated by perceived stress, further cementing its central role in sleep disturbances. Although improving family cohesiveness may be difficult through simple social interventions, targeted interventions toward increasing individual psychological security and developing coping strategies for effective mitigation of stress may yield notable downstream effects for promoting quality sleep. Sleep is, however, only one of many outcomes. Because stress is often accompanied by emotional arousal (Sterling et al, 1988), future research examining a more comprehensive model of specific stress consequences may help to elucidate where stress management may bear most benefit (Ye, Wu, et al., 2020; Ye, Zhou, et al., 2020; Ye, Wu, et al., 2021; Ye, Yang, et al., 2020).

### Limitations

Several limitations need to be considered for this study. Firstly, the cross-sectional nature of data collection limits inference of causality. Secondly, all variables were measured via self-report scales. Future research may opt to measure physiological readings of sleep quality to determine and establish stronger causal relations. Finally, COVID-19 has developed to different maturation stages across countries, with some experiencing delayed waves of outbreaks and others experiencing new outbreaks of different variants. Thus, further verification with samples from other countries may be needed.

## Conclusion

In summary, although further replication of the current results is still needed to make definitive claims, this study is an important step in examining how family cohesion relates to sleep disturbances symptoms among college students in China. As COVID-19 presents a myriad of stressors to college students, some being specific to COVID-19 (e.g., stress of infection) in addition to general stressors exacerbated by the current environment (e.g., financial stressors), it remains important to consider ways in which we may design interventions to tackle sleep issues. Interventions directly targeting one’s sense of security and stress may yield direct benefits to sleep quality while those targeting family cohesion may yield downstream benefits.

## Data Availability

The data and materials reported in this study are available from the first or corresponding author upon reasonable request.

## References

[CR1] Adams, G. C., Stoops, M. A., & Skomro, R. P. (2014). Sleep tight: Exploring the relationship between sleep and attachment style across the life span. *Sleep Medicine Reviews, 18*(6), 495–507. 10.1016/j.smrv.2014.03.002. 10.1016/j.smrv.2014.03.002 24721278

[CR2] Amaral AP, Soares MJ, Pinto AM, Pereira AT, Madeira N, Bos SC, Marques M, Roque C, Macedo A (2018). Sleep difficulties in college students: The role of stress, affect and cognitive processes. Psychiatry Research.

[CR3] Ayittey FK, Dzuvor C, Ayittey MK (2020). Updates on Wuhan 2019 novel coronavirus epidemic. Journal of Medical Virology.

[CR4] Aydin, B., & Oztutuncu, F. (2001). Examination of Adolescents’ Negative Thoughts, Depressive Mood, and Family Environment. *Adolescence, 36*(141), 77–77. 11407637

[CR5] Bannink R, Broeren S, Van de Looij Jansen PM, Raat H (2013). Relationship between quality of parent-child attachment, adverse life events and mental health. Multidisciplinary Sciences..

[CR6] Benham G, Charak R (2019). Stress and sleep remain significant predictors of health after controlling for negative affect. Stress and Health.

[CR7] Berg, K. A., Francis, M. W., Ross, K., & Spilsbury, J. C. (2021). Opportunities to improve sleep of children exposed to interpersonal violence: A social-ecological perspective. *Children and Youth Services Review, 127*, 106082. 10.1016/j.childyouth.2021.106082. 10.1016/j.childyouth.2021.106082 PMC9455662 36090582

[CR8] Billows, M., Gradisar, M., Dohnt, H., Johnston, A., McCappin, S., & Hudson, J. (2009). Family Disorganization, Sleep Hygiene, and Adolescent Sleep Disturbance. *Journal of Clinical Child & Adolescent Psychology, 38*(5), 745–752. 10.1080/15374410903103635. 10.1080/15374410903103635 20183658

[CR9] Bozorgmanesh K, Nazari AM, Zahrakar K (2016). Effectiveness of family therapy on its cohesion and flexibility. Journal of Holistic Nursing and Midwifery.

[CR10] Brand S, Beck J, Kalak N (2011). Dream recall and its relationship to sleep, perceived stress, and creativity among adolescents. Journal of Adolescent Health.

[CR11] Buysse DJ, Reynolds CF, Monk TH, Berman SR, Kupfer DJ (1989). The Pittsburgh sleep quality index: A new instrument for psychiatric practice and research. Psychiatry Research.

[CR12] Buzohre, E, L., Cheng, J., Liang, Y. M. (2018). Posttraumatic stress disorder and depression prevalence among enterprise employees after an accident disaster. *China Journal of Public Health, 34*(10), 1355–1359. 10.11847/zgggws1117242.

[CR13] Coe JL, Davies PT, Sturge-Apple ML (2017). The multivariate roles of family instability and interparental conflict in predicting children’s representations of insecurity in the family system and early school adjustment problems. Journal of Abnormal Child Psychology.

[CR14] Cohen S, Kamarck T, Mermelstein R (1983). A global measure of perceived stress. Journal of Health and Social Behavior.

[CR15] Cong Z, An LJ (2004). Developing of security questionnaire and its reliability and validity. Chinese Mental Health Journal.

[CR16] Cousins, J. C., Bootzin, R. R., Stevens, S. J., Ruiz, B. S., & Haynes, P. L. (2007). Parental involvement, psychological distress, and sleep: A preliminary examination in sleep disturbed adolescents with a history of substance abuse. *Journal of Family Psychology, 21*(1), 104–113. 10.1037/0893-3200.21.1.104. 10.1037/0893-3200.21.1.104 17371115

[CR17] Crespo C, Kielpikowski M, Pryor J, Jose PE (2011). Family rituals in New Zealand families: Links to family cohesion and adolescents’ well-being. Journal of Family Psychology.

[CR18] Cummings EM, Koss KJ, Davies PT (2015). Prospective relations between family conflict and adolescent maladjustment: Security in the family system as an explanatory mechanism. Journal of Abnormal Child Psychology.

[CR19] Cumsille PE, Epstein N (1994). Family cohesion, family adaptability, social support, and adolescent depressive symptoms in outpatient clinic families. Journal of Family Psychology.

[CR20] Donarelli Z, Dennis M, Kivlighan Jr, Allegra A (2016). How do individual attachment patterns of both members of couples affect their perceived infertility stress? An actor-partner interdependence analysis. Personality and Individual Differences.

[CR21] Fei LP, Shen QJ, Zheng YP, Zhao JP, Jiang SA, Wang LW (1991). Preliminary evaluation of Chinese version of FACES II and FES: Comparison of normal families and families of schizophrenic. Chinese Mental Health Journal.

[CR22] Gardner, K. A., & Cutrona, C. E. (2004). Social support communication in families. In Handbook of family communication (pp. 495–512). Lawrence Erlbaum Associates Publishers.

[CR23] Gerber M, Hartmann T, Brand S (2010). The relationship between shift work, perceived stress, sleep and health in Swiss police officers. Journal of Criminal Justice.

[CR24] Gupta, R., Grover, S., Basu, A., Krishnan, V., Tripathi, A., Subramanyam, A., Nischal, A., Hussain, A., Mehra, A., Ambekar, A., Saha, G., Mishra, K. K., Bathla, M., Jagiwala, M., Manjunatha, N., Nebhinani, N., Gaur, N., Kumar, N., Dalal, P. K., … & Avasthi, A. (2020). Changes in sleep pattern and sleep quality during COVID-19 lockdown. *Indian Journal of Psychiatry,**62*(4), 370–378. 10.4103/psychiatry.IndianJPsychiatry_523_20 10.4103/psychiatry.IndianJPsychiatry_523_20 PMC7597722 33165382

[CR25] Gunn, H. E., Troxel, W. M., Hall, M. H., & Buysse, D. J. (2014). Interpersonal distress is associated with sleep and arousal in insomnia and good sleepers. *Journal of Psychosomatic Research, 76*(3), 242–248. 10.1016/j.jpsychores.2013.11.010. 10.1016/j.jpsychores.2013.11.010 PMC4018775 24529045

[CR26] Gregory, A. M., Eley, T. C., O’Connor, T. G., Rijsdijk, F. V., & Plomin, R. (2005). Family influences on the association between sleep problems and anxiety in a large sample of pre-school aged twins. *Personality and Individual Differences, 39*(8), 1337–1348. 10.1016/j.paid.2005.06.008.

[CR27] Gregory, A. M., Caspi, A., Moffitt, T. E., & Poulton, R. (2006). Family Conflict in Childhood: A Predictor of Later Insomnia. *Sleep, 29*(8), 1063–1067.10.1093/sleep/29.8.1063. 10.1093/sleep/29.8.1063 16944675

[CR28] Hancock GR, Mueller RO (2010). The reviewer’s guide to quantitative methods in the social sciences. The reviewer’s guide to quantitative methods in the social sciences.

[CR29] Harvey AG (2002). A cognitive model of sleep disturbances. Behaviour Research and Therapy.

[CR30] Huang Y, Zhao N (2020). Generalized anxiety disorder, depressive symptoms and sleep quality during COVID-19 outbreak in China: A web-based cross-sectional survey. Psychiatry Research.

[CR31] Jin, Y. B., Lan, F., Sun, W. J. (2011). Cognitive models of sleep disturbances: A lecture. *Chinese Journal of Mental Health, 25*(7), 496–499. 1000–6729 (2011) 007–0496–04.

[CR32] Johnson DA, Billings ME, Hale L (2018). Environmental determinants of insufficient sleep and sleep disorders: Implications for population health. Current Epidemiology Reports.

[CR33] Johnson EO, Roth T, Breslau N (2006). The association of sleep disturbances with anxiety disorders and depression: Exploration of the direction of risk. Journal of Psychiatric Research.

[CR34] Kliewer W, Murrelle L, Prom E, Ramirez M, Obando P, Sandi L, del Carmen Karenkeris M (2006). Violence exposure and drug use in Central American youth: Family cohesion and parental monitoring as protective factors. Journal of Research on Adolescence.

[CR35] Lazarus RS, Folkman S (1984). Coping and adaptation.

[CR36] Lee S-Y, Wuertz C, Rogers R, Chen Y-P (2013). Stress and sleep disturbances in female college students. American Journal of Health Behavior.

[CR37] Li, S. W., Zhao Z. X., & Pan, J. Y. (2006). Consensus on definition, diagnosis and medication therapy of sleep disturbances. *Chinese Journal of Neurology, 39*, 141⁃143.

[CR38] Li, X. Y., Wei, X. Y., Chen, H. D., Gao, L. F., & Li, W. J. (2019). Relationship between perceived stress and perceived sleep quality: A dual-stage moderated mediation model among university students. *Chinese Journal of Clinical Psychology, 27*(02), 351–355. 10.16128/j.cnki.1005-3611.2019.02.029.

[CR39] Liu XC, Tang MQ, Hu L, Wang AZ, Wu HX, Zhao GF, Li WS (1996). The study on the reliability and validity of Pittsburgh Sleep Quality Index. Chinese Journal of Psychiatry.

[CR40] Mariani, R., Renzi, A., Di Trani, M., Trabucchi, G., Danskin, K., & Tambelli, R. (2020). The Impact of Coping Strategies and Perceived Family Support on Depressive and Anxious Symptomatology During the Coronavirus Pandemic (COVID-19) Lockdown. *Frontiers in Psychiatry, 11*, 1195. 10.3389/fpsyt.2020.587724. 10.3389/fpsyt.2020.587724 PMC7691226 33281647

[CR41] Masarik, A. S., & Conger, R. D. (2017). Stress and child development: A review of the Family Stress Model. *Current Opinion in Psychology, 13*, 85–90. 10.1016/j.copsyc.2016.05.008. 10.1016/j.copsyc.2016.05.008 28813301

[CR42] Maslow AH, Hirsh E, Stein M, Honigmann I (1945). A clinically derived test for measuring psychological security-insecurity. The Journal of General Psychology.

[CR43] Mitchell DB, Szczerepa A, Hauser-Cram P (2016). Spilling over: Partner parenting stress as a predictor of family cohesion in parents of adolescents with developmental disabilities. Research in Developmental Disabilities.

[CR44] McCarthy, S. (2020). ‘Don’t panic’ universities advise students in China coronavirus lockdown. South China Morning Post. https://www.scmp.com/news/china/society/article/3047585/dont-panic-universities-advisestudents-china-coronavirus.

[CR45] Olson DH (1986). Circumplex model VII: Validation studies and FACES III. Family Process.

[CR46] Olson, D. H., Portner, J., & Bell, R. (1982). *FACES II: Family adaptability and cohesion evaluation scales*. Unpublished manuscript, Family Social Science, University of Minnesota.

[CR47] Peltz, J. S., Rogge, R. D., & O’Connor, T. G. (2019). Adolescent sleep quality mediates family chaos and adolescent mental health: A daily diary-based study. *Journal of Family Psychology, 33*(3), 259–269. 10.1037/fam0000491. 10.1037/fam0000491 30589286

[CR48] Pinto J, van Zeller M, Amorim P, Pimentel A, Dantas P, Eusébio E, Neves A, Pipa J, Santa Clara E, Santiago T, Viana P, Drummond M (2020). Sleep quality in times of Covid-19 pandemic. Sleep Medicine.

[CR49] Prime, H., Wade, M., & Browne, D. T. (2020). Risk and resilience in family well-being during the COVID-19 pandemic. *American Psychologist, 75*(5), 631–643. 10.1037/amp0000660. 10.1037/amp0000660 32437181

[CR50] Qin, Y. H., Wan, X., Qu, S. S., & Chen, G. (2015). *Family cohesion and school belonging in preadolescence: Examining the mediating role of security and achievement goals*. SHS Web of Conferences (19). EDP sciences. 10.1051/shsconf/20151902004.

[CR51] Rojo-Wissar DM, Owusu JT, Nyhuis C, Jackson CL, Urbanek JK, Spira AP (2020). Parent–child relationship quality and sleep among adolescents: Modification by race/ethnicity. Sleep Health.

[CR52] Roth, N., Lev-Wiesel, R., & Shochat, T. (2019). “How do you sleep?” sleep in self-figure drawings of young adolescents in residential care facilities-An exploratory study. *Sleep Medicine,**60*,. 10.1016/j.sleep.2019.01.028 10.1016/j.sleep.2019.01.028 30795897

[CR53] Sadeh A, Raviv A, Gruber R (2000). Sleep patterns and sleep disruptions in school-age children. Developmental Psychology.

[CR54] Sher L (2020). COVID-19, anxiety, sleep disturbances and suicide. Sleep Medicine.

[CR55] Spilsbury, J. C., Patel, S. R., Morris, N., Ehayaei, A., & Intille, S. S. (2017). Household chaos and sleep-disturbing behavior of family members: Results of a pilot study of African American early adolescents. *Sleep Health, 3*(2), 84–89. 10.1016/j.sleh.2016.12.006. 10.1016/j.sleh.2016.12.006 PMC5373486 28346162

[CR56] Sterling, P., Eyer, J., Fisher, S., & Reason, J. (1988). *Handbook of life stress, cognition and health. John Wiley & Sons.*

[CR57] Tang WJ, Wang G, Hu T, Dai Q, Xu J, Yang Y (2018). Mental health and psychological problems among Chinese left-behind children: A cross-sectional comparative study. Journal of Affective Disorders.

[CR58] Tsai KM, Dahl RE, Irwin MR, Bower JE, McCreath H, Seeman TE, Almeida DM, Fuligni AJ (2018). The roles of parental support and family stress in adolescent sleep. Child Development.

[CR59] Vazsonyi AT, Jiskrova GK, Ksinan AJ (2018). Sleep, low self-control, and deviance: Direct and indirect links across immigrant groups and socioeconomic strata. Journal of Adolescence.

[CR60] Wang, C., & Zhao, H. (2020). The impact of COVID-19 on anxiety in Chinese university studentS. *Frontiers in Psychology,**11*, 1168. 10.3389/fpsyg.2020.01168 10.3389/fpsyg.2020.01168 PMC7259378 32574244

[CR61] Yan YW, Liu MY, Tang XD, Lin RM (2010). On the relationship among stress response, coping and sleep quality. Advances in Psychological Science.

[CR62] Yang YZ, Huang HT (2003). An epidemiological study on stress among urban residents in social transition period. Chinese Journal of Epidemiology.

[CR63] Ye, B., Lei, X., Yang, J., Byrne, P. J., Jiang, X., Liu, M., & Wang, X. (2021). Family cohesion and social adjustment of Chinese university students: The mediating effects of sense of security and personal relationships. *Current Psychology, 40*(4), 1872–1883. 10.1007/s12144-018-0118-y.

[CR64] Ye, B., Wu, D., Im, H., Liu, M., Wang, X., & Yang, Q. (2020). Stressors of COVID-19 and stress consequences: The mediating role of rumination and the moderating role of psychological support. *Children and Youth Services Review, 118,* 105466. 10.1016/j.childyouth.2020.105466. 10.1016/j.childyouth.2020.105466 PMC7515821 32994656

[CR65] Ye, B., Zhou, X., Im, H., Liu, M., Wang, X. Q., & Yang, Q. (2020). Epidemic rumination and resilience on college students’ depressive symptoms during the COVID-19 pandemic: The mediating role of fatigue. *Frontiers in Public Health, 8*, 10.3389/fpubh.2020.560983. 10.3389/fpubh.2020.560983 PMC7755644 33363075

[CR66] Ye, Z., Yang, X., Zeng, C., Wang, Y., Shen, Z., Li, X., & Lin, D. (2020). Resilience, social support, and coping as mediators between COVID-19-related stressful experiences and acute stress disorder among college students in China. *Applied Psychology: Health and Well-Being, 12*(4), 1074–1094. 10.1111/aphw.12211. 10.1111/aphw.12211 PMC7405224 32666713

[CR67] Ye, B., Fan, N., Im, H., Chen, Z., Liu, M., Wang, X., & Yang, Q. (Working Paper). Family cohesion and trust: The mediating role of psychological stress responses of COVID-19 and the moderating role of stress mindset.

[CR68] Ye, B., Wu, D., Wang, P., Im, H., Liu, M., Wang, X., & Yang, Q. (2021). COVID-19 Stressors and Poor Sleep Quality: The Mediating Role of Rumination and the Moderating Role of Emotion Regulation Strategies. *International Journal of Behavioral Medicine.*10.1007/s12529-021-10026-w. 10.1007/s12529-021-10026-w PMC8477722 34581977

[CR69] Zhang, H. (2020). The Influence of the Ongoing COVID-19 Pandemic on Family Violence in China. *Journal of Family Violence, *1–11. 10.1007/s10896-020-00196-8. 10.1007/s10896-020-00196-8 PMC7473410 32921903

